# Pulling Back the Curtain: Improving Reviews in Environmental Health

**DOI:** 10.1289/ehp.1002691

**Published:** 2010-08

**Authors:** Tracey J. Woodruff, Patrice Sutton

**Affiliations:** Program on Reproductive Health and the Environment, Department of Obstetrics, Gynecology, and Reproductive Sciences, University of California, San Francisco, Oakland, California, E-mail: woodrufft@obgyn.ucsf.edu

The environment is increasingly being recognized as an important driver of human health and the weight of the evidence is sufficiently high, thus warranting timely preventative action (Diamanti-Kandarakis et al. 2009; President’s Cancer Panel 2010; Woodruff et al. 2008). However, it is a challenge to keep abreast of the deluge of scientific literature linking the environment to health. The volume and highly variable quality of data, as well as the barrage of bits and pieces of information, can leave the public and policy makers (and scientists) confused and overwhelmed as they try to grapple with the meaning behind the information. A perusal through PubMed on any of the hot topics in environmental health [e.g., bisphenol A, phthalates, PBDEs (polybrominated diphenyl ethers)] finds numerous studies on each topic. Each of us tries to sort through the accumulating evidence to understand its meaning, whether at the most personal level (e.g., should I give my baby water using a sippy cup made from polycarbonate plastic) to a more global level (e.g., should a pesticide be banned from use). Too much noise can diffuse rather than coalesce our common perception of the signal and undermine our capacity to act wisely.

Thus, reviews of scientific evidence are a critical step toward speeding the incorporation of the science into action to prevent harm. Reviews assemble and synthesize the evidence across studies to inform an overall conclusion about state-of-the-science knowledge. Simple in concept, but challenging in application, lessons can be learned from the clinical field, which has grappled with a parallel need to incorporate the meaning of the science in a systematic and timely manner into beneficial patient treatment decisions and prevention methods.

Historically, the clinical field relied largely on a system of expert reviews on which to base treatment decisions (Rennie and Chalmers 2009). However, starting in the 1970s, the role of expert reviews began to be questioned for a number of reasons (e.g., potential bias of experts, timeliness of information); this led to the development of systematic approaches that would use rigorous, transparent, and explicit methodology to evaluate a clearly formulated question. Landmark papers published in the clinical literature, such as Antman et al. (1992), showed that reviews based solely on expert opinion “did not work” and demonstrated the superiority of systematic reviews for patient outcomes (Rennie and Chalmers 2009).

The need for a comparable approach to reviews is equally compelling in the field of environmental health science because of the large number of studies and because they are as subject to bias as those in the clinical sciences. Notably, the influence of financially conflicted sources of funding is well recognized in the clinical world (Rennie 2010), and widely used examples of systems that utilize best practices for weighing and communicating the strength of the scientific evidence (West et al. 2002) prohibit sponsorship by any commercial source or sources [i.e., Cochrane Reviews (Higgins and Green 2006)] or recommend that the quality of potentially conflicted evidence be downgraded when evaluated [i.e., Grading of Recommendations, Assessment, Development and Evaluation (GRADE) (Guyatt et al. 2008)]. Similar concerns have been raised in the environmental health literature (President’s Cancer Panel 2010) and warrant similar attention.

However, although the clinical sciences point the way, these systems are not fully transferable to environmental health science because of differences in the types of evidence generally available and how decisions to expose populations and individuals are made. For example, in the clinical setting, *in vivo*, *in vitro*, and human experimental evidence combined with an analysis of risks and benefits have informed human exposure decisions prior to the the entry of the substances into the marketplace. Systematic reviews in the clinical sciences proceed from this evidence and context.

In stark contrast, population exposure to exogenous substances in the environment typically occurs before regulatory scrutiny of a compound and in the absence of risk–benefit analysis, because of the current regulatory structure for governing manufactured chemicals. Ethical considerations virtually preclude experimental human data from the environmental health evidence stream, so we must rely on *in vitro* and *in vivo* studies for early warnings of adverse effects and on human observational studies to assess the nature and extent of the damage.

To rapidly move the best science into improved health outcomes, we need to apply the pertinent lessons and embrace the continued challenges of the clinical sciences. To that end, we are collaborating with 22 scientists and clinicians from the United States and Europe to craft the Navigation Guide, a systematic and transparent methodology that proceeds from GRADE but reflects the differences in evidence and decision contexts. Professional societies, health care organizations, government agencies, and other potential guideline developers working with toxicologists can use the Navigation Guide to craft consistent and timely recommendations to improve patient and population health outcomes.

Finally, the science of systematic reviews in the clinical sciences has evolved to be a relatively well-resourced and respected academic field in its own right. Currently, the science of environmental health science review and synthesis is largely relegated to regulatory and other government agencies, and few resources are allocated to the science of interpretation. The rising prominence of the environment as a key determinant of health requires that we devote concordant effort to primary scientific discovery and to the building upon and synthesis of the research through the systematic and transparent reviews. The reviews in this issue of *Environmental Health Perspectives* are a welcome step in the right direction.

## Figures and Tables

**Figure:**
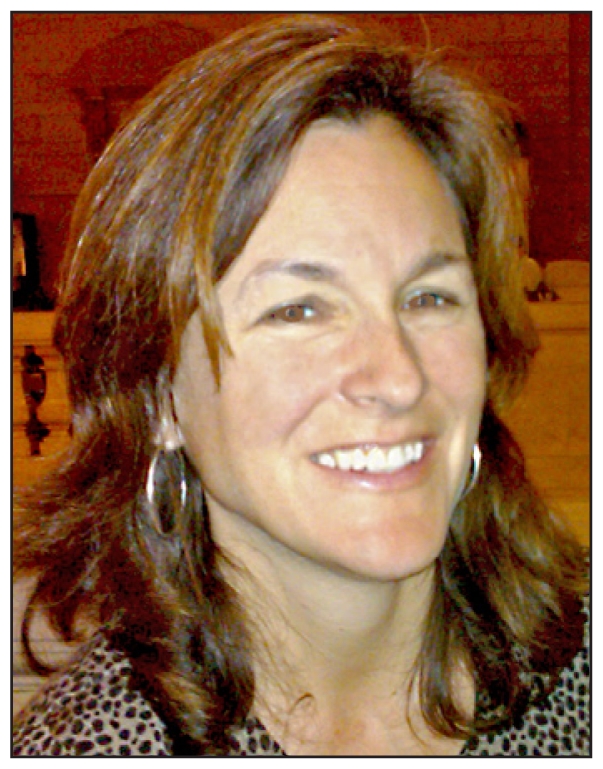
Tracey J. Woodruff

**Figure:**
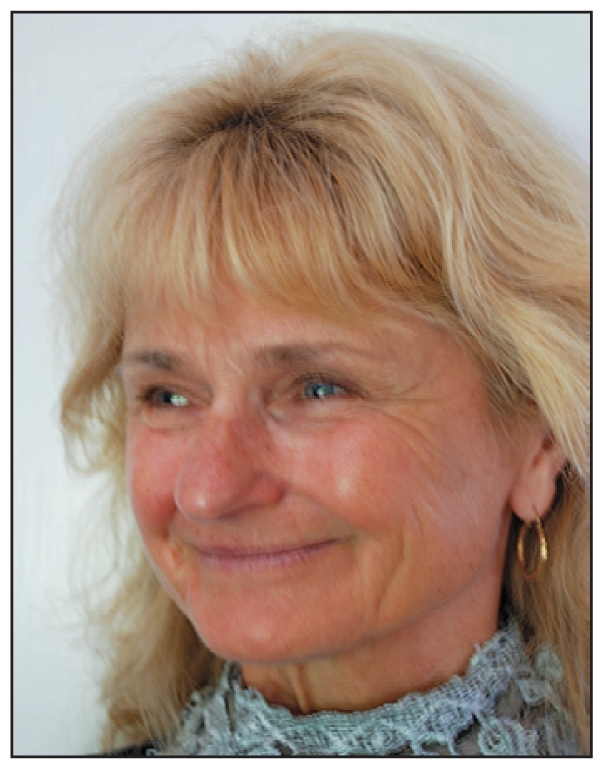
Patrice Sutton

## References

[b1-ehp.118-a326] Antman EM, Lau J, Kupelnick B, Mosteller F, Chalmers TC (1992). A comparison of results of meta-analyses of randomized control trials and recommendations of clinical experts. Treatments for myocardial infarction. JAMA.

[b2-ehp.118-a326] Diamanti-Kandarakis E, Bourguignon JP, Giudice LC, Hauser R, Prins GS, Soto AM (2009). Endocrine-disrupting chemicals: an Endocrine Society scientific statement. Endocr Rev.

[b3-ehp.118-a326] Guyatt GH, Oxman AD, Vist GE, Kunz R, Falck-Ytter Y, Alonso-Coello P (2008). GRADE: an emerging consensus on rating quality of evidence and strength of recommendations. BMJ.

[b4-ehp.118-a326] Higgins JPT, Green S (2006). Cochrane Handbook for Systematic Reviews of Interventions 4.2.6.

[b5-ehp.118-a326] President’s Cancer Panel (2010). Reducing Environmental Cancer Risk: What We Can Do Now.

[b6-ehp.118-a326] Rennie D (2010). Integrity in scientific publishing. Health Serv Res.

[b7-ehp.118-a326] Rennie D, Chalmers I (2009). Assessing authority. JAMA.

[b8-ehp.118-a326] West S, King V, Carey TS, Lohr KN, McKoy N, Sutton SF (2002). Systems to rate the strength of scientific evidence. Evid Rep Technol Assess (Summ).

[b9-ehp.118-a326] Woodruff TJ, Carlson A, Schwartz JM, Giudice LC (2008). Proceedings of the Summit on Environmental Challenges to Reproductive Health and Fertility: executive summary. Fertil Steril.

